# MeinteR: A framework to prioritize DNA methylation aberrations based on conformational and cis-regulatory element enrichment

**DOI:** 10.1038/s41598-019-55453-8

**Published:** 2019-12-16

**Authors:** Andigoni Malousi, Sofia Kouidou, Maria Tsagiopoulou, Nikos Papakonstantinou, Emmanouil Bouras, Elisavet Georgiou, Georgios Tzimagiorgis, Kostas Stamatopoulos

**Affiliations:** 1 Lab. of Biological Chemistry, School of Medicine, Aristotle University of Thessaloniki, Thessaloniki, Greece; 2 Institute of Applied Biosciences, Centre for Research and Technology Hellas, Thessaloniki, Greece; 3 Lab. of Hygiene, Social-Preventive Medicine & Medical Statistics, School of Medicine, Aristotle University of Thessaloniki, Thessaloniki, Greece

**Keywords:** Cancer genomics, Computational biology and bioinformatics

## Abstract

DNA methylation studies have been reformed with the advent of single-base resolution arrays and bisulfite sequencing methods, enabling deeper investigation of methylation-mediated mechanisms. In addition to these advancements, numerous bioinformatics tools address important computational challenges, covering DNA methylation calling up to multi-modal interpretative analyses. However, contrary to the analytical frameworks that detect driver mutational signatures, the identification of putatively actionable epigenetic events remains an unmet need. The present work describes a novel computational framework, called MeinteR, that prioritizes critical DNA methylation events based on the following hypothesis: critical aberrations of DNA methylation more likely occur on a genomic substrate that is enriched in cis-acting regulatory elements with distinct structural characteristics, rather than in genomic “deserts”. In this context, the framework incorporates functional cis-elements, e.g. transcription factor binding sites, tentative splice sites, as well as conformational features, such as G-quadruplexes and palindromes, to identify critical epigenetic aberrations with potential implications on transcriptional regulation. The evaluation on multiple, public cancer datasets revealed significant associations between the highest-ranking loci with gene expression and known driver genes, enabling for the first time the computational identification of high impact epigenetic changes based on high-throughput DNA methylation data.

## Introduction

Basic and applied research on DNA methylation (DNAm) have been revolutionized with the advent of single-base resolution and genome-wide assays. Along with these advancements, a wide range of bioinformatics methods has been developed to address the computational complexities of high-throughput analyses. These methods are generally classified in two categories: (a) those focusing on the core analysis pipeline that transforms raw array-based^1,2^ or sequencing data to DNAm calls^3,4^, and (b) those implementing downstream analyses^5–11^, e.g. cell mixture proportions, age calculators, differential analysis, visualization, association with gene expression and phenotypic data, pathway enrichment analyses, genomic architecture mappings etc. Furthermore, several frameworks provide comprehensive solutions by either integrating existing tools from both categories in user-friendly pipelines^12–16,^ or by interpreting DNAm events with respect to the enrichment of diverse types of colocalized regulatory elements^11^.

While significant progress has been made on improving DNAm calling methods and enriching the types of interpretative analyses^17^, the need to computationally identify the most critical aberrations is still poorly addressed. The main origins of this deficiency are: (a) the plethora of differentially methylated sites (DMS) that are usually identified from high-throughput experiments^18^; (b) the dynamics and tissue specificity of DNAm events; (c) modest interpretability, as aberrant DNAm is usually observed in poorly annotated, non-coding regions^18^; and (d) unlike genomic studies, lack of efficient analytical frameworks that detect critical events^19^. To this end, a computational framework that could address the above issues would constitute a significant advancement in interpretative DNAm analyses.

In particular, recent studies highlight the benefits of encompassing DNAm data in computational frameworks that deal with driver event detection. For example, a beta mixture model was proposed for the detection of important methylation-driven genes in cancer by integrating methylome and gene expression data^20,21^. A similar data- driven pathway method identified pan-cancer genes by integrating DNAm, copy number variation and gene expression data^22^. In the same context, a functional interaction network developed to prioritize cancer genes from multi-omics data, including DNAm^23^. The above methods share certain common features: (a) driving events do not solely derive from DNAm profiles; (b) aberrant DNAm is inferred at gene level by averaging DNAm levels at promoters, intronic and exonic regions; and (c) pathway enrichment from each -omics modality is used to classify driver genes.

In addition, these studies do not encounter an important regulator of epigenetic modifications, that is the genomic substrate underlying driver events. The contribution of specific positional (e.g. cis-acting regulatory elements) and compositional features (e.g. CpG islands, k-mers) in DNAm has been highlighted in multiple research studies. For example, a set of cis-acting, methylation-prone and methylation-resistant motifs were identified that increase the predictive power of the DNAm detection methods^24^. Other studies elaborated further on: (a) the role of context-dependent DNAm as instructor for gene regulation^25^, (b) associated DNAm with the presence of ENCODE’s regulatory elements^26^, and (c) developed computational tools that accurately predict DNAm levels, based on context-based features^27–29^.

The role of the genomic context is also supported by several studies associating the presence of particular regulatory elements with DNAm. For example, dual-specificity of transcription factors is related with variable binding affinity in methylated and unmethylated forms of a CpG sequence^30,31^. As transcription factor binding sites are identified by position-specific dependencies among nucleotides it is important to specify whether putative bindings are potentially inhibited or promoted when co-localized with methylated CpG sites (CpGs), implying an indirect role of DNAm in regulatory processes^32^. In the same context, DNA sequences that fold into G-quadruplexes were found to be less prone to CpG methylation, while increased DNAm is depleted in these structures^33^, and might change protein binding to quadruplex-forming DNA segments during transcriptional regulation, particularly in aging^34,35^.

Although less clearly demonstrated, non-canonical hairpin structures formed by palindromic sequences could potentially regulate methylation-mediated processes by becoming resistant to DNAm^36^. Furthermore, short sequences neighboring methylated cytosines in palindromic sequences were found to attract protein-DNA binding^37^. Other DNA helix elements, such as local geometric features (minor/major groove, propeller twist etc.), alter their shapes in the presence of DNAm, and could probably affect protein-DNA binding, subsequently leading to transcriptional activation or silencing^38^. In this context, computational methods could corroborate or deputize limited experimental data by predicting local structural changes of the double helix induced by DNAm that may successively alter protein-DNA binding affinity^39^. DNAm also exhibits distinguishable positional patterns in constitutive and alternative splicing^40–43^. CpGs that are located on the exon-intron junctions exhibit increased DNAm levels, contrary to the neighboring intronic regions^44,45^. Considering these complexities, further analyzed by Machado *et al*.^46^, it is evident that the genomic substrate could provide valuable insights in deciphering the impact of aberrant DNAm events.

Herein, we present a computational framework, called MeinteR, that identifies putatively functional DNAm sites based on the following hypothesis: aberrant DNAm that occurs on a genomic substrate enriched in cis-regulatory and conformational elements is more likely to trigger methylation-mediated transcriptional events than differential DNAm observed in genomic “deserts”. In this context, MeinteR builds genomic signatures of DMS and identifies critical loci where aberrant DNAm might have a greater effect on phenotype expression, using a linear function of the elements enrichment, called genomic index. With three use cases and extensive comparisons, we show that MeinteR provides an efficient means to decode complex associations between DNAm aberrations and gene expression deregulation.

## Results

**Figure 1:**
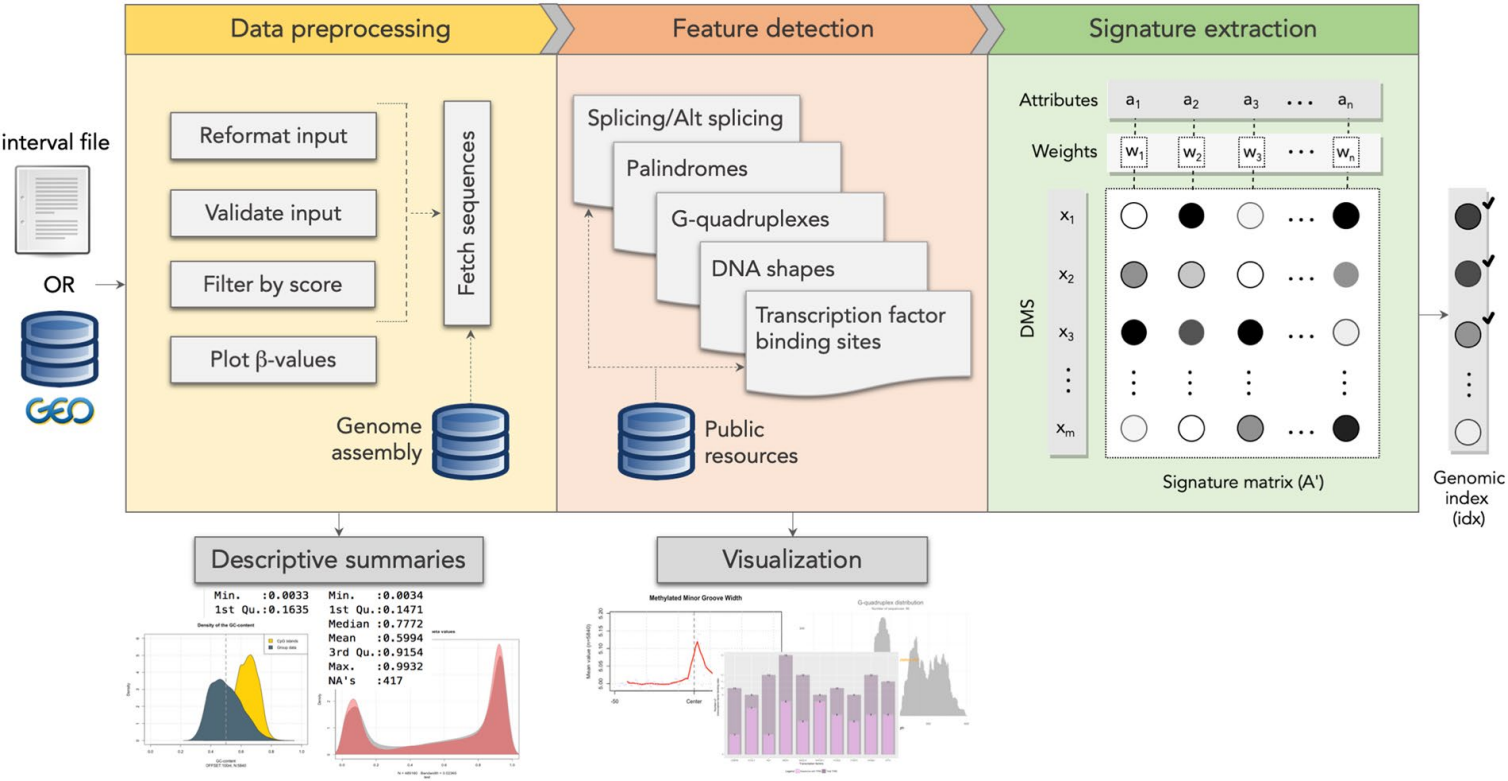
Overview of the MeinteR workflow. Input data, either interval files or GEO data series, are pre-processed and genomic sequences are obtained from human genome assembly (*data preprocessing* panel). Plotting functions and summary statistics supplement the data preprocessing module. Fetched DNA sequences are then analysed with respect to the abundance of the incorporated features through the corresponding MeinteR functions that export tabular and graphical outputs (*feature detection* panel). The identification of transcription factor binding sites and splicing elements is based on reference data that are automatically retrieved from relevant public resources. The last module (*signature extraction* panel) builds a matrix of genomic signatures per CpG site and the genomic index is calculated based on user-defined weighting schemes. The genomic signature of each DNAm is a numerical vector containing the abundance of each feature, multiplied by a user-defined weighting factor. The genomic index is a non-negative real number that is calculated using the linear mixture of the values in the signature vector.

MeinteR is a computational framework consisting of three modules (Fig. 1). Briefly, the *data preprocessing* module contains functions for loading, validation, reformatting and filtering of DNAm data that are exported from BeadChip arrays or next-generation sequencing platforms. The *feature detection* module implements a set of functions for batch sequence retrieval and enrichment analysis of the incorporated features. Finally, the *signature extraction* module builds genomic signatures of the candidate sites and implements a ranking scheme. The functionality of each module is described in Methods.

MeinteR is a software package that primarily builds genomic signatures of epigenetic aberrations and identifies critical events in high-throughput datasets. The modularity of the software components enables a wide variety of applications in multiple settings as shown in the following use cases. Use case 1 shows a differential analysis of tumor/normal samples using only G-quadruplexes, and use case 2 builds genomic signatures of tumor/normal samples using the complete set of conformational and cis-regulatory elements. Use case 3 implements the primary goal of the framework that is to export the genomic index of aberrantly methylated sites and to associate the genomic index with differential gene expression. In these use cases, DNAm data are retrieved from Gene Expression Omnibus (GEO)^47^ and The Cancer Genome Atlas (TCGA)^48^ and for most differential analyses we applied a stringent threshold (Δβ > = 0.3, *p* < 0.01 and FDR < 0.01) to avoid the detection of false positive DMS sites and improve the quality of downstream analysis towards biological interpretation of the results. Notably, the objective of these use cases is to provide pre-configured examples on public datasets, rather than to interpret the biological findings. These use cases can be easily adapted to other research applications using custom configurations as regards to the composition of the feature set and weighting scheme. MeinteR provides supplementary functions to further annotate the input data in terms of the CpG/G + C content and *β* value distribution. These functions, coupled with various filtering parameters embedded in each core function, can be used towards comprehensive and accurate characterization of the input data.

### Use Case 1: Genome-wide association of G-quadruplexes with DNAm using public breast cancer datasets

**Figure 2:**
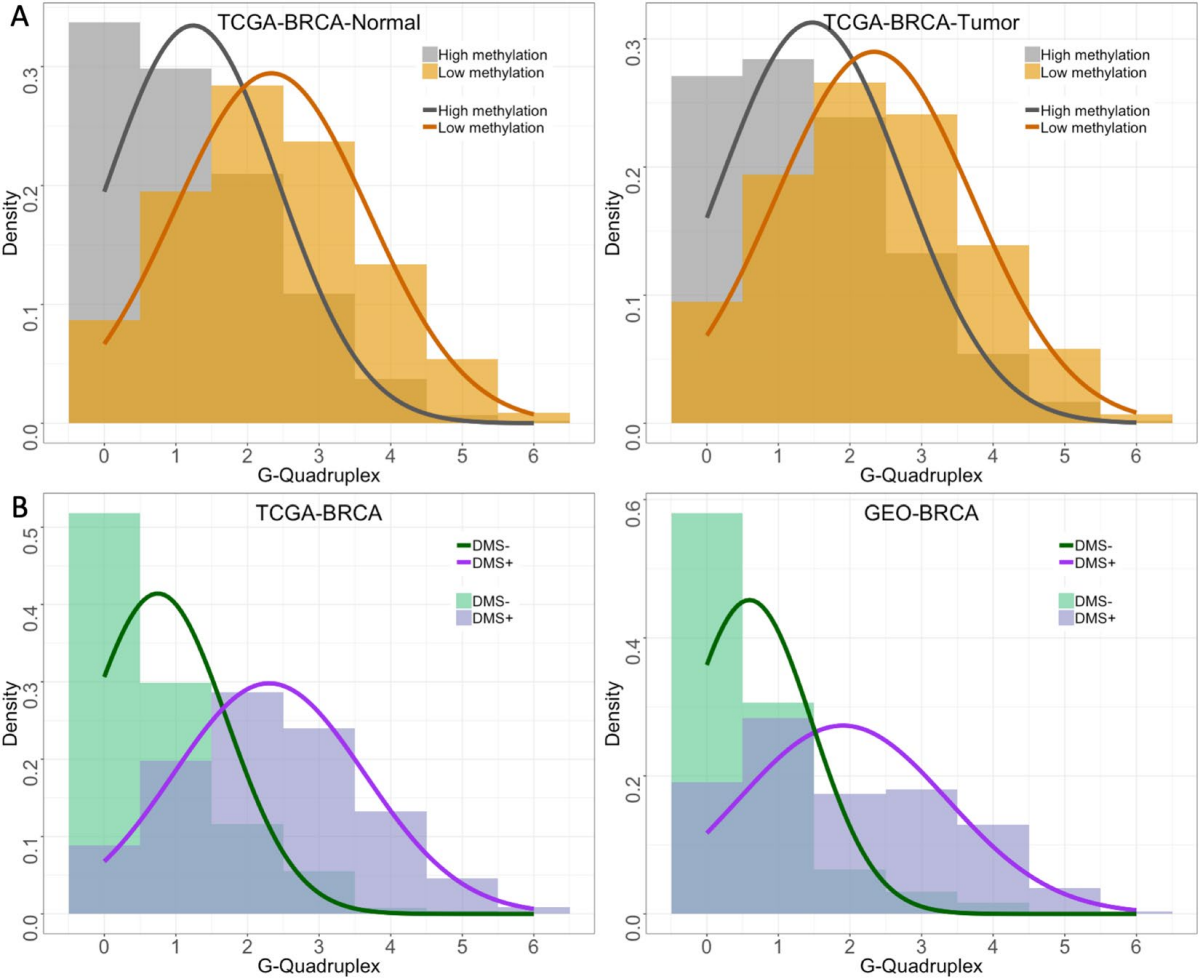
Histograms of the G-quadruplex density in breast cancer. (**A**) Comparison of low and highly methylated sites in matched normal tissue (left) and primary breast tumor (right) retrieved from TCGA. (**B**) Comparison of hypermethylated (DMS^+^) and hypomethylated (DMS^−^) sites in TCGA and GEO breast cancer samples (left and right panel, respectively). The curves correspond to normal distributions projected over the histograms.

To demonstrate the applicability in revealing associations between DNAm and particular genomic features, we used MeinteR to investigate DNAm resistance in sequences that fold in G-quadruplex structures^33^. First, we downloaded TCGA HumanMethylation450 array data from 91 breast cancer patients with matched primary tumor and normal tissue samples^47^. Then, we calculated the mean DNAm levels per sample group using the beta (β) values of the interrogated CpGs. *β* values range from 0 to 1 and are calculated by the formula *β* = Intensity of the methylated probe/(Intensity of the unmethylated probe + Intensity of the methylated probe + 100). For each sample group, we identified G-quadruplex structures in sequences centered at unmethylated and methylated sites, with *β* ≤ 0.1 and *β* ≥ 0.9, respectively. Batch analysis of normal samples revealed a statistically significant two-fold increase (two-tailed t-test, *p* < 0.001) of G-quadruplex frequency at unmethylated sites compared to methylated sites (Fig. 2A, left-hand side). Similarly, as shown in Fig. 2A (right-hand side), in primary breast tumor samples G-quadruplex-forming sequences are less frequently observed in regions neighboring highly methylated sites (two-tailed t-test, *p* < 0.001).

We further evaluated the propensity of G-quadruplex structures to co-localize with sites that significantly lower their DNAm level in cancer (hypomethylated, DMS^−^) and vice versa, sites that exhibit a significant decrease in their DNAm level in cancer (hypermethylated, DMS^+^) with ($|{\rm{\Delta }}\beta |\ge 0.3$, *p *< 0.01 and FDR < 0.01). Differential analysis of the cancer/normal breast pairs identified 3,981 DMS^+^, and 1,869 DMS^−^. Both datasets were randomly subsampled to 1,000 DMS and scanned for putative G-quadruplexes. Figure 2B on the left side shows a three-fold increase of G-quadruplex frequency in DMS^+^ compared with DMS^−^ (two-tailed t-test, *p* < 0.001). This observation is inline with the results shown in Fig. 2A, since DMS^+^ mostly involves low methylated sites in normal samples due to the bimodal distribution of the *β* values and vice versa.

To validate these results, we followed the same procedure on HumanMethylation450 DNAm data obtained from 80 breast cancer and 40 normal samples that are deposited to GEO (GSE66695 data series). *β* values were averaged on each sample group and differentially analyzed, resulting in 293 DMS^+^ and 62 DMS^−^ (|$|{\rm{\Delta }}\beta |\ge 0.3$, *p* < 0.01, FDR < 0.01). Then, we estimated the frequency of G-quadruplex forming sequences in 100nt regions centered at DMS. The histogram in Fig. 2B (right-hand side) shows that most DMS^−^ sequences lack G-quadruplex structures (mean G4: 0.597, s.d. = 0.878), while most DMS^+^ are co-localized with at least one G-quadruplex structure (mean G4: 1.901, s.d. = 1.462). The difference is statistically significant (two-tailed t-test, *p* < 0.001) and in accordance with the outcome of the TCGA data analysis (Fig. 2B, left). Overall, the results of both breast cancer datasets dictate a protective role of G-quadruplex structures against DNAm that apparently characterizes DNAm patterns independently of the cell type and phenotype.

To further validate these findings, we performed the same analysis on six other cancer types using TCGA datasets. Fig. S1 (Supplementary File 1) shows the results that further validate the same findings. Overall, the results dictate a protective role of G-quadruplex structures against DNAm that apparently characterizes DNAm patterns independently of the cell type and phenotype. This observation needs further elaboration that is beyond the scope of the present work.

### Use Case 2: Evaluation of genomic signatures on cancer DNAm profiles

In this  use case, we determined the enrichment of the incorporated conformational and regulatory elements, in order to assess the contribution of the genomic substrate to DNAm alterations in cancer. To this end, we built the genomic signatures of nine cancer types using DNAm datasets deposited to GEO^48^. Each cancer dataset contains DNAm data from tumor and normal samples. Table 1 lists the mean values, standard deviations and statistical evaluation of each feature in the hypo- and hypermethylated subsets. In addition, each subset is characterized with respect to the CpG/G + C content in all cancer types. As expected, sites that are hypermethylated in cancer are located in more G + C/CpG-rich regions than DMS^−^. Overall, the abundance of DMS varies, depending on the assay. In addition, the number of DMS^+^ and DMS^−^ differs significantly in all cancer types, yet not in the same way. The abundance of G-quadruplex structures in DMS^+^ and DMS^−^ sites (Table 1, *G4* column) is statistically different in eight out of nine cancer types (*p* < 0.01). Similarly, the frequency of palindromic sequences (Table 1, *Pals* column) in DMS^+^ and DMS^−^ sites is significantly different in six out of nine cancer types, while transcription factor binding sites exhibit also important differences. Transcription factor binding sites exhibit statistically significant differences in one out of nine cancer types (Table 1, *TFBS* column), while for conserved human/mouse/rat transcription factors the statistical significance is observed in five cancer types (Table 1, *cTFBS* column). Alternative splicing events exhibit mixed profusion and statistically significant results in two out of six datasets. Among four conformational changes, only minor groove width and propeller twist seem to affect or to be affected by differential DNAm in a small subset of cancer data (Supplementary File 1, Table S1), that is partially consistent with recent non-cancer-specific analyses^39^.

**Table 1:** *p*-values of methylation-mediated features in cancer DNAm data obtained from BeadChip GEO data series.

GEO ID	Cancer Data Series	Samples	Assay	DMS^(+/−)^	G + C/ΟΕ CpG content	G4		Pals		Alt. Spl.		TFBS		cTFBS		Ref
						mean(sd)	*p*-val	mean(sd)	*p*-val	*p*-val	mean(sd)	*p*-val	mean(sd)	*p*-val	mean(sd)	
GSE42752	Colorectal adenocarcinoma	22/22	HM450k	2,028	0.68/0.83	2.90 (1.33)	**<10**^**−3**^	9.65 (4.63)	**0.002**	—	0.07 (0.25)	0.065	5.96 (5.81)	0.23	0.35 (1.17)	71
				49	0.55/0.52	1.51 (1.10)		7.53 (3.11)			—		9.25 (4.19)		0.2 (0.91)	
GSE54503	Hepatocellular carcinoma	66/66	HM450k	1,227	0.69/0.85	2.87 (1.37)	**<10**^**−3**^	10.43 (5.03)	**<10**^**−3**^	**<10**^**−3**^	0.07 (0.25)	0.143	5.61 (5.07)	**<10**^**−3**^	0.38 (1.16)	51
				7,490	0.53/0.52	1.18 (1.19)		6.9 (3.06)			0.01 (0.08)		6.62 (6.52)		0.08 (0.5)	
GSE85464^*^	Gastric adenocarcinoma	19/19	HM450k	161	0.65/0.78	2.57 (1.37)	**<10**^**−3**^	8.95 (4.94)	**<10**^**−3**^	—	0.08 (0.27)	**0.002**	5.06 (4.82)	**<10**^**−3**^	0.4 (1.13)	72
				353	0.53/0.57	1.25 (1.24)		6.9 (3.4)			—		11.25 (14.29)		0.1 (0.66)	
GSE25093	Head & NeckSC carcinoma	91/18	HM27k	16	0.60/0.68	1.69 (1.13)	0.188	8.12 (4.96)	0.56	0.423	0.06 (0.25)	0.481	12 (12.19)	0.28	0.81 (2.74)	73
				83	0.53/0.4	1.29 (1.04)		7.06 (3.18)			0.02 (0.15)		9.32 (11.9)		0.16 (0.63)	
GSE32866^*^	Lung adenocarcinoma	28/27	HM27k	175	0.66/0.82	2.45 (1.35)	**<10**^**−3**^	9.78 (4.62)	**<10**^**−3**^	0.941	0.04 (0.20)	0.443	5.73 (5.1)	0.174	0.63 (1.56)	74
				23	0.53/0.35	1.17 (1.27)		5.26 (2.07)			0.04 (0.21)		9.67 (8.96)		0.13 (0.34)	
GSE37754^#^	Breast cancer	62/10	HM450k	152	0.59/0.69	1.95 (1.39)	**<10**^**−3**^	7.25 (2.72)	0.466	—	0.03 (0.18)	0.546	5.67 (9.54)	0.36	0.24 (0.92)	75
				49	0.48/0.39	1.02 (1.13)		6.9 (3.1)			—		4.83 (3.19)		0.1 (0.42)	
GSE26989	Ovarian cancer	41/10	HM27k	613	0.56/0.49	1.54 (1.34)	**0.001**	7.12 (3.52)	0.099	0.304	0.09 (0.29)	0.751	7.98 (9.56)	**0.008**	0.32 (1.17)	76
				1,160	0.53/0.38	1.31 (1.21)		6.84 (3.11)			0.08 (0.27)		7.57 (7.93)		0.18 (0.69)	
GSE109402	Medulloblastoma	33/5	EPIC	14,120	0.55/0.43	1.22 (1.19)	**<10**^**−3**^	7.23 (3.25)	**0.0095**	**0.002**	0.04 (0.19)	0.121	6.1 (5.76)	**0.0099**	0.16 (0.72)	77
				56,444	0.48/0.32	0.95 (1.09)		6.86 (3.09)			0.01 (0.12)		7.83 (8.29)		0.08 (0.53)	
GSE61441	Renal cell carcinoma	46/46	HM450k	86	0.60/0.74	1.97 (1.52)	**<10**^**−3**^	8.2 (3.53)	**0.002**	0.36	0.03 (0.18)	0.95	4.64 (2.62)	**0.002**	0.36 (0.94)	78
				129	0.50/0.35	1.15 (1.21)		6.78 (2.97)			0.02 (0.12)		5.69 (5.84)		0.09 (0.64)	

*DMS*^+^ and *DMS*^*−*^ columns contain the number of hypermethylated and hypomethylated sites (DMS) respectively, for various cancer datasets (*Cancer Data Series*) and BeadChip assays. Maximum 1,000 sites were analyzed. *p*-values (*p-val* columns) are calculated with t-test and Wilcoxon test for non-normal distributions in addition to the mean values and standard deviations for each feature (*mean(sd)* columns). Column *G + C*/*OE CpG content* contains the G + C content and the observed/expected (OE) ratio of the CpG frequencies. Putative transcription factor binding sites (*TFBS* column) were identified only in DMS sequences located in promoters. The *samples* column contains the number of tumor/normal samples included from each data series (matched pairs when numbers are identical). *p*-values are not shown when no DMS co-localized with alternative splicing events (*Alt. Spl* column). ^*^|Δβ| > 0.25, ^#^transformed M-values, |Δβ| > 0.20. *G4*: G-quadruplexes, *Pals*: Palindromes, *cTFBS*: Conserved TFBS, SC: squamous cell.

In this use case, MeinteR automates the investigation of complex associations between epigenomic and genomic features across different cancer types. The results demonstrate a clear association between DNAm profiles and the genomic substrate that should be further elaborated and interpreted in the context of disease-specific analyses, as different outcomes may be attributed to the design, assay, and the experimental protocol of each study.

### Use Case 3: Associating genomic DMS signatures with gene expression

The objective of this use case is to appraise the association between the genomic index and differential gene expression. First, we used MeinteR to download a GEO dataset for which both DNAm and gene expression data of the same samples are available. We used a set of 24 non-muscle invasive bladder cancer and matched normal samples (BLCA/GSE37817)^49^. To build expression profiles at gene level we applied GEO2R^48^, and calculated the differential expression levels between cancer and control samples, using the binary logarithmic fold change. To analyze DNAm data, we used MeinteR in order to: (a) import *β* values of all samples, and (b) calculate mean *β* values per group. DMS were finally mapped to expression data and the level of differential DNAm was correlated with the expression levels of the mapped genes. To validate the results, we performed the same steps on processed TCGA Illumina HiSeq expression data from primary bladder urothelial carcinomas and normal tissues.

**Figure 3:**
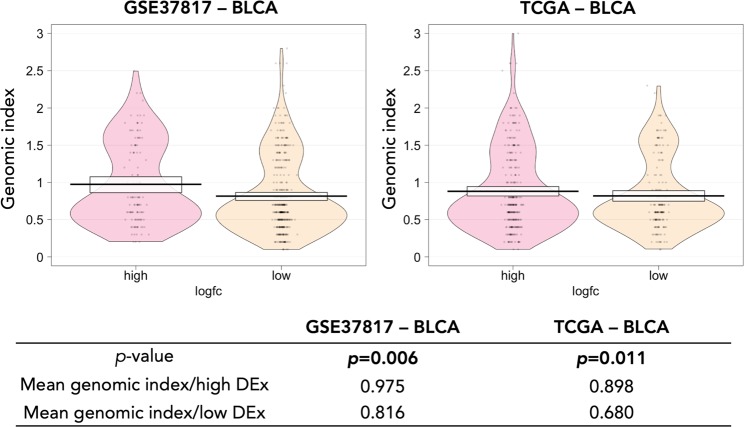
Smoothed density curves corresponding to the distribution of the genomic index in high differentially expressed genes (DEx) and low DEx in bladder cancer (BLCA). Horizontal black lines correspond to the mean genomic index and white boxes show the 95% bayesian highest density intervals.

Differential DNAm analysis resulted in 1,474 probes that are more frequently located in “open sea” regions and less frequently to CpG islands. Fig. S2 (Supplementary File 1) illustrates the distribution of the genomic index in different regions relative to CpG islands. Probes located in CpG islands exhibit statistically significant differences of the genomic index and increased mean genomic index against all other regions (Shelf: *p*-val = 0.02, Shore: *p*-val < 0.001, Open sea: *p*-val < 0.001). None of the pairwise differences between shelves, shores and open sea probes were found statistically significant. DMS located in CpG islands were further annotated based on their position relative to gene regions. The density plots in Fig. S2 show that most CpG island DMS are located in 5′UTR and first exons, while the probes located in 200nt upstream transcription start sites (TSS200) have the highest mean genomic index. As expected, DMS^+^ are more often located in genes that decrease their expression level, while DMS^−^ are spatially linked with genes that increase their expression with significant statistical difference (*p* < 0.001) for both BLCA datasets (Supplementary File 1, Fig. S3). To demonstrate the relevance of the genomic substrate in prioritizing critical DNAm events, we used MeinteR to assess whether differential gene expression is associated with higher genomic index. First, we calculated the genomic index of all DMS by assigning equal weights to the incorporated feature set. Figure 3 shows that among all aberrantly methylated sites, higher absolute differential expression is observed in sequences with increased genomic index. The differences are statistically significant for both BLCA datasets from TCGA and GEO (*p* < 0.05), implying that the effect of differential DNAm in gene expression is probably modulated by the underlying genomic elements involved in the transcriptional regulation. The linear regression models for genomic index and logarithmic fold-change pairs are shown in Fig. S4 (Supplementary File 1).

### Comparison with epigenetic markers and driver mutational signatures of hepatocellular carcinoma

To evaluate the competency of our approach, we performed comparisons with software tools and computational methods that identify driver events in two settings: (a) comparison between the genomic index and Δ*β* values to find the best-fitting metric for identifying epigenetic markers, and (b) correlations of the highly prioritized sites with known mutation-based driver genes and epimarkers.

**Figure 4:**
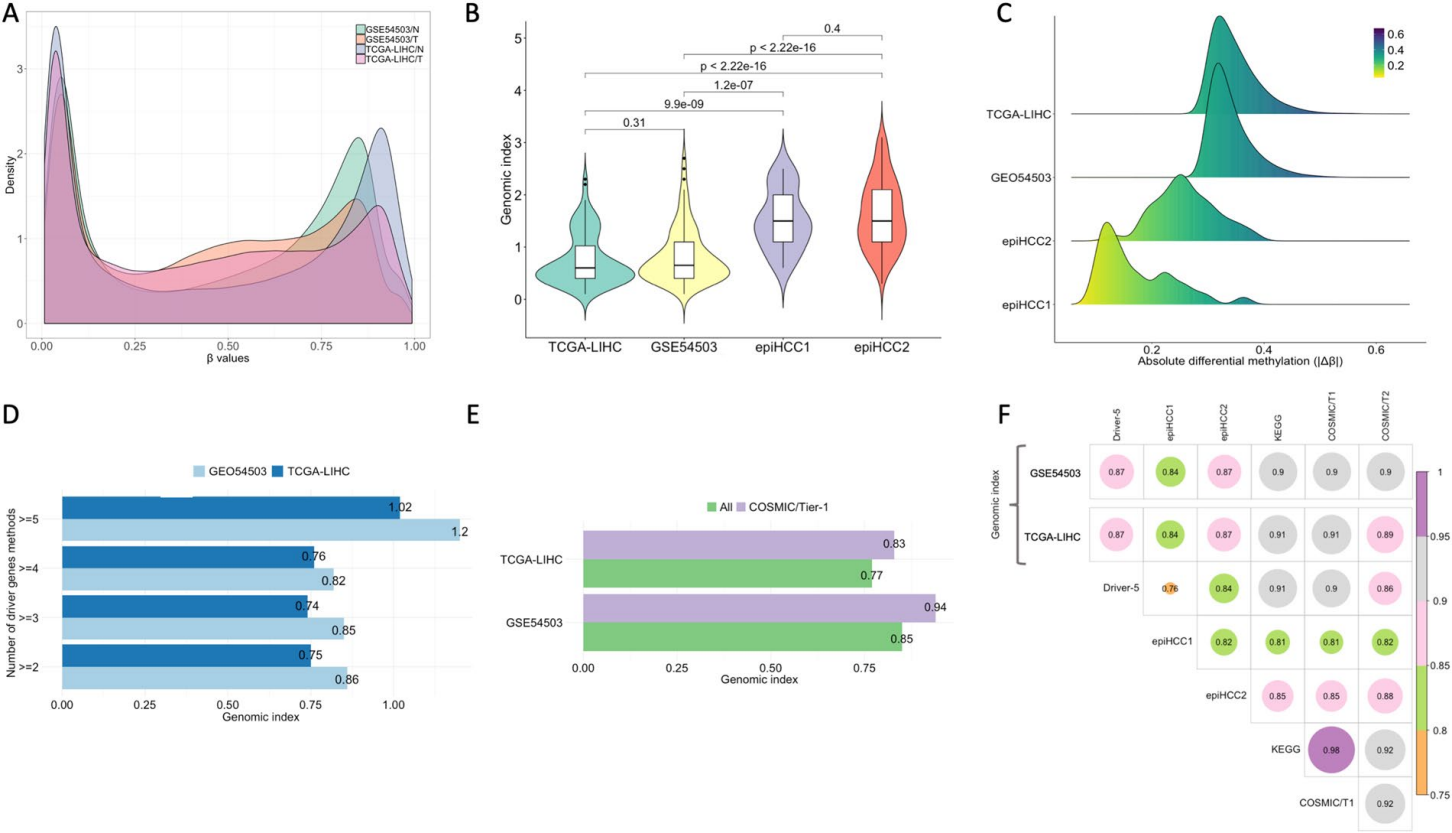
(**A**) Mean *β* value densities of the tumor(T)/normal(N) samples included in two public HCC datasets (TCGA-LIHC, GSE54503). (**B**) Smoothed density curves of the genomic index distributions and *p*-values (two-tailed t-test) for all pairwise comparisons between TCGA-LIHC/GSE54503 datasets and epimarkers (epiHCC1, epiHCC2). (**C**) Differential *β* value density of the TCGA-LIHC/GSE54503 datasets and epimarkers (epiHCC1, epiHCC2). (**D**) Mean genomic index of aberrantly methylated sites |Δβ| ≥ 0.3) in driver genes that have been detected by at least two to five computational methods. (**E**). Mean genomic index of all TCGA-LIHC and GSE54503 differentially methylated sites compared with the genomic index of COSMIC Tier-1 (T1) cancer gene census. (**F)**. Semantic similarity plot correlating highly-ranked sites of the TCGA-LIHC and GSE54503 datasets, according to MeinteR, with the COSMIC T1, Tier 2 (T2) Gene Census, KEGG HCC pathway, Driver-5 genes and epigenetic markers (epiHCC1, epiHCC2).

DNAm data from hepatocellular carcinoma (HCC) were used to assess the overall performance. First, we downloaded TCGA level 3 HumanMethylation450 data from 50 primary HCC and matched normal pairs and built genomic signatures of the 13,153 DMS ($|\Delta \beta |\ge 0.3$, *p* < 0.01, FDR     < 0.01), using equal-weighted attributes. Overlaps with palindromes, G-quadruplexes and conserved transcription factors were analyzed within 100nt region adjacent to each DMS. All sequences were scanned for transcription factors that unveil differential binding on DNAm targets in HepG2 cell line, using the curated data of the MEDReaders database^50^. To validate the results, we additionally used MeinteR to build genomic signatures of 8,717 DMS ($|\Delta \beta |\ge 0.3$, *p* < 0.01, FDR < 0.01) exported from 66 matched HCC and adjacent non-tumor tissues (GSE54503 data series)^51^, using the same configuration. The datasets exhibit similar bimodal *β* value distributions for normal and tumor samples (Fig. 4A). The list of all critical DMS according to our ranking scheme is provided in Supplementary File 2.

To assess the efficiency of our method, we performed comparisons with known HCC markers that have been identified using DNAm data. Specifically, we found two probe-sets corresponding to: (a) 33 high-confidence epimarkers (epiHCC1) predicted by Zheng *et al*.^52^, and (b) 109 HCC epimarkers (epiHCC2) identified by Cheng *et al*.^53^. For each reference probe-set, we calculated the enrichment of the genomic substrate using MeinteR. The genomic index of the epiHCC1 and epiHCC2 markers was estimated using the same configuration as for the TCGA-LIHC and GSE54503 datasets and is listed in Supplementary File 3. Figure 4B shows that epiHCC1 and epiHCC2 markers exhibit significantly more enriched genomic substrate (epiHCC1: mean g.index = 1.52, s.d. = 0.57, epiHCC2: mean g.index = 1.62, s.d. = 0.65), compared with the genomic index of all aberrantly methylated sites identified in the TCGA-LIHC and GSE54503 datasets (TCGA-LIHC: mean g.index = 0.77, s.d. = 0.52, GSE54503: mean g.index = 0.85, s.d. = 0.53). The differences of the genomic index levels are statistically significant for the TCGA-LIHC/epiHCC1 (*p* = 9.9e-09) and GSE54503/epiHCC1 (*p* = 1.2e-07) pairs and, as expected, not important for the TCGA-LIHC/GSE54503 comparison (*p* = 0.31). In accordance, the genomic index differs significantly for the TCGA-LIHC/epiHCC2 (*p* < 2.22e-16) and GSE54503/epiHCC2 (*p* < 2.22e-16) pairs. The genomic index of the epiHCC1 and epiHCC2 markers does not exhibit statistically significant differences (*p* = 0.4).

Cheng *et al*.^53^ identified six epiHCC2 markers in four genes (*NEBL, FAM55C, GALNT3*, and *DSE*) that are hypermethylated exclusively in HCC. Interestingly, these HCC-specific diagnostic biomarkers have higher genomic index, compared to all 109 epiHCC2 markers (mean genomic index 2.03 vs.1.62, respectively), and more than two-fold higher genomic index than the average genomic index of all DMS identified in the TCGA/LIHC and GSE54503 datasets. Notably, the epimarkers of both methods would have been missed, if the Δ*β* level was used for selecting the most critical sites, since the differential DNAm level of the epimarkers (Fig. 4C) is lower (epiHCC1: mean |Δ*β|* = 0.17, s.d. = 0.07, epiHCC2: mean |Δ*β|* = 0.26, s.d. = 0.05) than the threshold commonly set in cancer-specific analyses |Δ*β|* ≥ 0.3). These results further support the relevance of the genomic index as an important criterion to prioritize actionable epigenetic markers compared with the usage of the Δ*β* level.

Next, we pursued the evaluation of our method with respect to known driver cancer genes, motivated by previous studies that revealed significant associations between mutations in driver cancer genes with DNAm alterations^54–56^. First, we built a set of candidate driver genes based on the number of detection methods that report their causality in HCC, using DriverDB^57^. DriverDB^57^ is a database that provides access to common driver genes that are computationally identified by 15 mutation-based methods. We assumed that more accountable driver genes are those reported by multiple driver detection methods and built the genomic signatures of driver genes with different levels of evidence. Accordingly, Driver-5 genes i.e. driver genes shared by at least five methods are more reliable than driver genes detected by two methods. Using these reference mutation-based data, we sought to compare the genomic index of low and high confident gene lists reported for HCC. The analysis of both TCGA-LIHC and GSE54503 datasets shows that driver genes identified by at least five methods (Driver-5) are associated with clearly higher genomic index than those with poorer evidence (Fig. 4D). The same procedure was applied on the gene set that is included in the COSMIC Cancer Gene Census (CGC, Tier 1)^58^. Compared with the entire set of DMS of TCGA-LIHC and GSE54504 datasets, those topologically linked with COSMIC CGC driver genes are on average associated with higher genomic index (Fig. 4E).

Finally, we compared MeinteR with relevant methods that detect driver genes/markers based on mutational and epigenetic HCC signatures. For this analysis, we additionally obtained the list of KEGG genes that are involved in the HCC pathway (hsa05225) and performed pairwise comparisons, in order to estimate the Wang’s semantic distance between Disease Ontology terms^59^ and the best-max average combination method^60^. The correlation plots in Fig. 4F show the semantic similarity levels of the highly-ranked TCGA-LIHC and GSE54503 genes with known driver genes and epimarkers. The comparison between MeinteR and the consensus mutation-based driver genes (*Driver-5*) shows that MeinteR exhibits similar semantic correlation with the KEGG and COSMIC genes and slightly better semantic correlation with COSMIC Tier 2 genes. These results are obtained using both TCGA and GEO datasets. Finally, the highly-ranked genes prioritized by our method are evidently better correlated with KEGG and COSMIC genes than epiHCC1 and epiHCC2 markers. As expected, the highest correlation level is observed between COSMIC CGC Tier 1 and KEGG pathway genes, while the epiHCC1/Driver-5 comparison has the lowest correlation level.

## Discussion

The role of DNAm in disease onset and progression has been extensively studied, particularly in cancer (reviewed by Chatterjee *et al*.^19^). Although epigenome aberrations are frequently observed in most cancer types, recent studies have shown that small sets of aberrantly methylated sites are able to discriminate cancer subtypes^54^ and to predict drug response^61^, posing computationally challenging questions on which of the epigenetic alterations are functionally key events in cancer. MeinteR consolidates our knowledge on methylation-modulating mechanisms enabling, for the first time, the identification of high-impact epigenetic alterations, under the prism of conformational and cis-regulatory element enrichment, quantified by the genomic index as a linear function of the feature abundance. The genomic index does not explicitly dictate the presence of protective regions or regions prone to transcriptional changes, yet higher values imply a greater incidence of methylation-modulated, functional elements, that might play a critical role in transcriptional events. In this context, MeinteR is better described as an approach that implements “upstream” biological interpretation, as it incorporates features associated with potential causes of the DNAm events, as opposed to the “downstream” biological interpretation that quantifies the effect of DNAm events on biological pathways^17^.

In comparison with other epigenetic driver detection methods, MeinteR differs in both the research hypothesis and methodology. First, MeinteR identifies the most influential DNAm sites, rather than driver DNAm alterations. The latter imply the presence of causal relationships between driver and passenger epigenetic alterations that are not essentially relevant with the genomic substrate. Second, most computational methods entail the integration of multi-omics data to identify driver events, e.g. gene expression, DNAm and copy number variations^20–22^. MeinteR relies exclusively on DNAm data enabling faster and straight-forward interpretative analyses of high-throughput experiments. The evaluation results demonstrate that aberrant DNAm sites, co-localized with putative conformational and cis-regulatory elements, are better correlated with known cancer drivers, suggesting a potential role in transcriptional regulation with significant diagnostic and therapeutic implications.

MeinteR is an open-source R package, easily applicable to TCGA and GEO data analyses, enabling case-by-case configuration of the incorporated features and weighting scheme. In addition, MeinteR is valuable in improving the accuracy of imputation methods, as it has been shown that the prediction of CpG methylation levels based only on neighboring CpG sites is suboptimal, especially in sparsely assayed genomic regions^26^. Equally important, MeinteR incorporates functions that are time-effectively applied in genome-wide DNAm datasets, with no special hardware requirements. In this respect, our contribution is inline with the FAIR principles^62^ (i.e. *Findable* – *Accessible*; as it is publicly available in a reference software repository – *Interoperable*; as it has been implemented in R, an open source environment, and exploits data and software from reference third-party repositories – *Reusable*; since besides its public availability, it is also accompanied with detailed documentation and comprehensive examples of use), fostering transparency and reproducibility of the source code.

Overall, with MeinteR we aim to provide the basis for exploratory and explanatory analyses related with development, aging, cancer and other biological processes and diseases complementing the interpretation of DNAm alterations, beyond local architecture annotations and pathway enrichments and with potential usability in developing predictive models for identifying disease subtypes and response to treatments.

## Methods

### Module 1: Data preprocessing

MeinteR’s functions are applied on bed-formatted chromosomal interval files containing the coordinates of each DMS, and the corresponding score values. These files are retrievable by tools performing differential DNAm analyses, such as limma^9^, RnBeads^63^, minfi^64^, ChAMP^16^, Bicycle^65^ etc. Alternatively, MeinteR is able to fetch array-based and sequencing-based data from GEO^48^ and to automatically build valid data files. In case of array platforms, such as Illumina’s BeadChip HumanMethylation27, HumanMethylation450 and MethylationEPIC, MeinteR splits samples in two subsets, according to a predefined annotation file that contains the list of sample identifiers and the corresponding group e.g. normal/tumor, pre-/post-treatment. Then, Δ*β* values are calculated and valid interval files are generated (Supplementary File 1, Fig. S5). Sequencing data from whole genome bisulfite-sequencing (WGBS), reduced representation bisulfite sequencing (RRBS) and targeted bisulphite-based experiments contain the number of methylated reads and read depth information per CpG site. MeinteR fetches sequencing data for each sample, filters data based on the read depth and chromosome, and builds interval files containing DNAm level per site, as a fraction of cytosine-reporting reads vs. the total number of mapped reads (example usage on WGBS, RRBS data is available on the software’s vignette). Besides data loading, the preprocessing module contains a set of functions for the validation of data values and format, *M* to *β* value conversion, as well as plotting and filtering functions.

### Module 2: Feature detection

MeinteR calculates the abundance of methylation-mediated features in variable-length sequences centered at each CpG target, using a set of functions as described below (Fig. 1).

#### Transcription factor binding motifs

MeinteR identifies putative binding sites of: (a) conserved transcription factors in human/mouse/rat alignments and (b) human transcription factors included in the JASPAR’s core collection (version 2018)^66^. Conserved factors and their ~5.8 million genome-wide binding loci are retrieved from the corresponding track of the UCSC Table Browser. The intersection of the binding loci and the genomic coordinates of the regions flanking each DMS is exported and comparatively visualized against the expected frequency. For the detection of JASPAR’s profile matrices, MeinteR uses the scanning algorithm implemented in TFBSTools^67^, in order to identify high-scoring matches between transcription factor profile matrices and DMS in user-defined sequence offset. To speed-up the analysis of large datasets, searches can be narrowed-down to promoters or CpG islands. In addition, MeinteR allows users to select a list of transcription factors, among hundreds available, and perform targeted enrichment analyses, excluding the “noisy” binding sites.

#### Palindromes and G-quadruplex structures

Palindromic sequences are detected using Biostrings^68^. First, MeinteR retrieves sequences of variable length centered at DMS and scans for palindromic regions based on user-defined arm lengths and in-between loop sizes. The identification of potential quadruplex-forming sequences is implemented using the pqsfinder algorithm^69^. As for palindromes, MeinteR retrieves genomic sequences corresponding to the coordinates of each DMS expanded by a user-defined offset and performs batch detection of G-quadruplex structures. The output of both functions includes summary and verbose reports of the detected readouts that are subsequently used to build genomic signatures.

#### Splice sites and alternative splicing events

MeinteR enables batch analyses of splicing-related events by: (a) detecting putative 5′ and 3′ splice sites, and (b) matching known alternative splicing events to the CpG coordinates. Generally, splice junctions and their short neighboring sequences are characterized by species-specific conserved motifs. In this work, motifs are described as position-specific weight matrices, following the definition of donor and acceptor sites by Shapiro & Senapathy^70^. Then, short sequences adjacent to DMS are searched for these matrices, using the same scanning method that is applied for transcription factor binding site detection^67^. The detection of overlapping alternative isoforms is based on known alternative splicing events available by the UCSC Table Browser. MeinteR calculates the frequency of different alternative splicing events overlapping DMS data and builds graphical reports of the observed and expected frequency in the human reference genome.

#### Conformational DNA features

To determine putative conformational DNA changes caused by DNAm, MeinteR uses the methyl-DNAshape algorithm^39^. For each DNAm site, the respective function retrieves short sequences of user-defined length adjacent to DMS in batches and uses methyl-DNAshape to calculate minor groove width, roll, propeller and helix twist in the unmethylated and methylated context. The difference between the two states is evaluated and the statistical significance of each DNA shape is calculated using Wilcoxon tests.

### Module 3: Signature extraction

The third module aggregates genomic features at each CpG site and: (a) constructs a signature matrix, and (b) performs multi-variate ranking to identify putatively actionable sites as shown below (Fig. 1).

#### Genomic signature matrix

The genomic signature of each DMS is assembled in a matrix containing at least one of the incorporated genomic features. Let ${x}_{i}\in X=\{{x}_{1},{x}_{2},\,\ldots ,\,{x}_{m}\}$ be a set of *m* DMS sites and ${a}_{i}\in A=\{{a}_{1},{a}_{2},\,\ldots ,\,{a}_{n}\}$ is the list of *n* attributes, i.e. genomic features associated with each CpG site. The attributes are of different scaling and data types and treated accordingly, in order to operate on the same scale. Splicing-related observations i.e. putative donor, acceptor sites and co-localized alternative splicing events are joined into logical values representing the incidence of at least one feature. Similarly, conformational changes are quantified as logical variables that are positive, when at least one DNA shape alteration is statistically significant (*p* < 0.05). Finally, the abundance of G-quadruplex structures, palindromic sequences and transcription factor binding sites (conserved, putative human JASPAR core collection 2018) are normalized to fit (0,1) scale. Overall, depending on the feature *a*_*j*_, a mapping function *m*_*j*_ is applied for each attribute *j*, where ${a^{\prime} }_{j}={m}_{j}({a}_{j})$ and the genomic signature matrix ${A}^{\text{'}}$ is built that tabulates the mapped attribute values *a*′_*ij*_ for each *x*_*i*_.

#### Multi-variate ranking

Given a signature matrix *A*′, the final step is to rank each DMS *x*_*i*_ based on the genomic index *idx*_*i*_, with ($id{x}_{i}\in {{\mathbb{N}}}^{+}$). For each *x*_*i*_, *idx*_*i*_ is defined by the weighted sum of all attributes *a*′_*ij*_. For example, if *w*_*j*_ is the positive weight of the *j*^*th*^ DMS attribute, *x*_*i*_ is defined by the sum of the weighted attribute values of *x*_*i*_, i.e.: $id{x}_{i}=\sum _{j=1}^{n}{w}_{j}{a}_{ij},i=1,2,\,\ldots ,\,m$. The output data are exported in an *m*-length vector of genomic index values quantifying the enrichment of the incorporated elements at each CpG site (genomic signature).

## Supplementary information

Supplementary file 1

Supplementary file 2

Supplementary file 3

## Data Availability

All data generated or analyzed during this study are included in this published article (and its Supplementary Information Files). MeinteR is an R package available under the GNU General Public Licence v3. The source code and binaries can be found at https://github.com/andigoni/MeinteR. The repository contains also documentation of the respective R package including a manual, a package tutorial with examples and the source code of the three use cases.
