# Supplementary material for: MeinteR: A framework to prioritize DNA methylation aberrations based on conformational and cis-regulatory element enrichment
Source: Sci Rep. 2019 Dec 16;9:19148. doi: 10.1038/s41598-019-55453-8 (PMC6915744; doi:10.1038/s41598-019-55453-8)
Supplement: Supplementary file 1 [file 41598_2019_55453_MOESM1_ESM.pdf]

**Supplementary Tables and Figures**

# MeinteR: A framework to prioritize DNA methylation aberrations based on conformational and cis-regulatory element enrichment

A. Malousi, S. Kouidou, M. Tsagiopoulou, N. Papakonstantinou, E. Bouras, E. Georgiou, G. Tzimagiorgis, K. Stamatopoulos

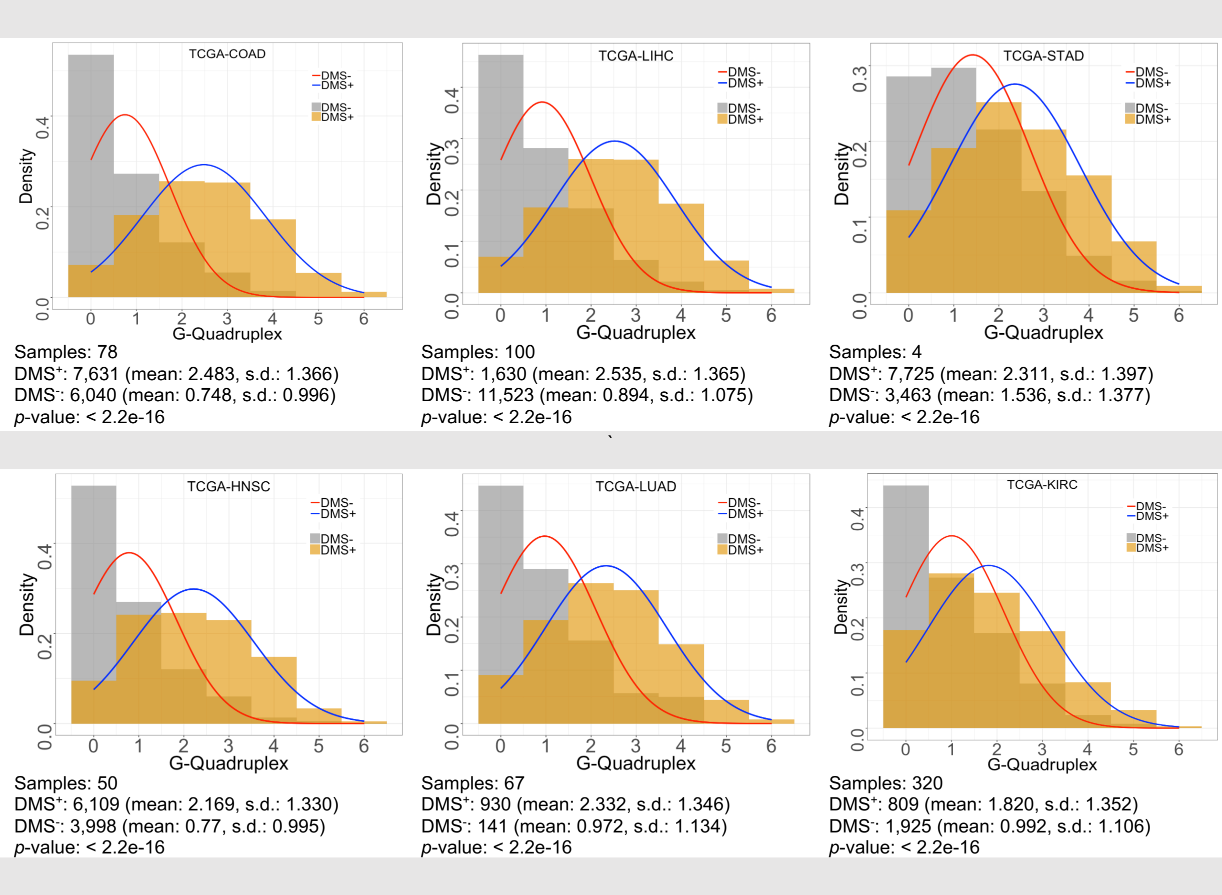


**Figure S1.** Histograms of the G-quadruplex density in six TCGA cancer types. The red and blue curves correspond to the normal distributions projected over the histograms. The number of matched tumor/normal samples, the number of DMS and the statistical evaluation (two-tailed t-test) are calculated in each panel.

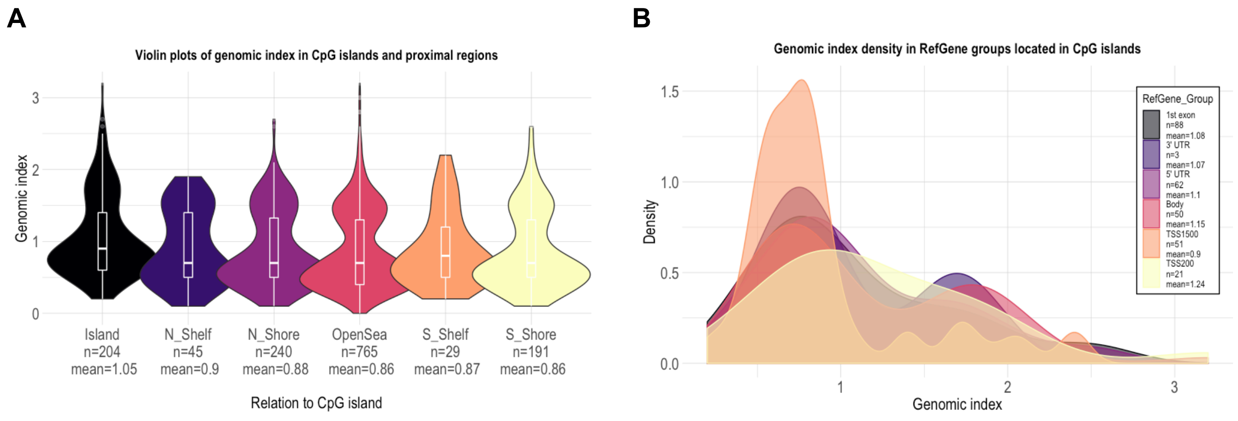


**Figure S2**. A. Violin plots of the genomic index and mean values corresponding to BLCA DMS in different regions relative to CpG islands. N_Shores(S_Shores): 2kb upstream(downstream) from the ends of the CpG islands, N_Shelf(S_Shelf): 2-4kb upstream(downstream) from the ends of the CpG islands. B. Density plots of the genomic index and mean values corresponding to BLCA DMS located in relative gene regions of CpG islands.

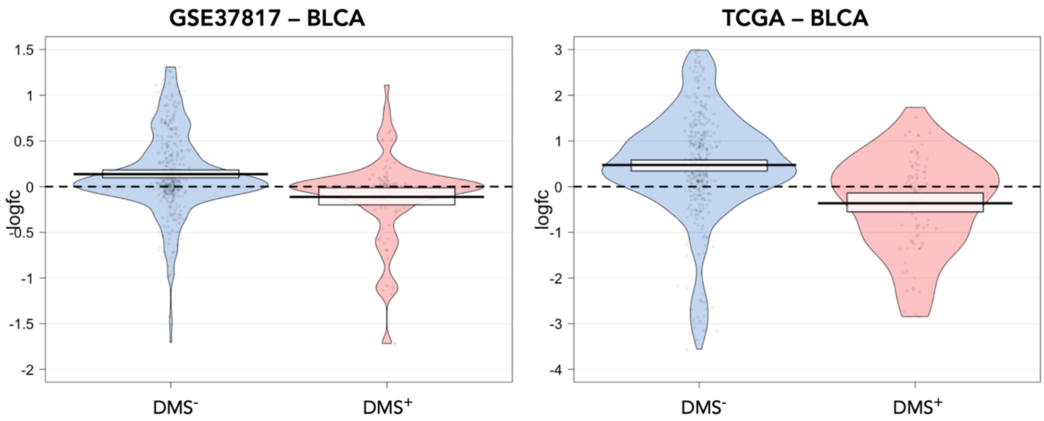


| | GSE37817 – BLCA | TCGA – BLCA |
|---|---|---|
| *p*-value | ***p*<0.0001** | ***p*<0.0001** |
| Mean logfc/DMS+ | -0.112 | -0.364 |
| Mean logfc/DMS- | 0.137 | 0.474 |

**Figure S3.** Boxplots illustrating the logarithmic fold change (logfc) of the expressed genes associated with hypomethylated DMS (DMS-) vs hypermethylated DMS (DMS+) in two bladder cancer datasets. The embedded table contains the *p*-values and mean logfc of the corresponding gene expression levels in the associated hyper- and hypomethylated sites.

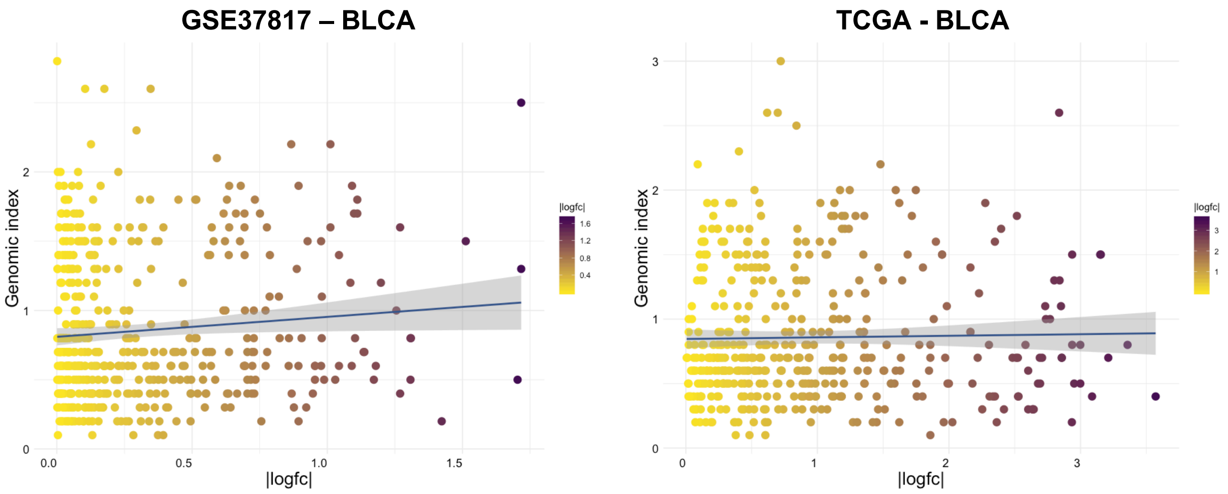


**Figure S4.** Scatterplots of the genomic index vs. absolute fold-change (*logfc*) of the BLCA expression levels calculated from the expression profiling of the GSE37817 data series (left) and TCGA (right).

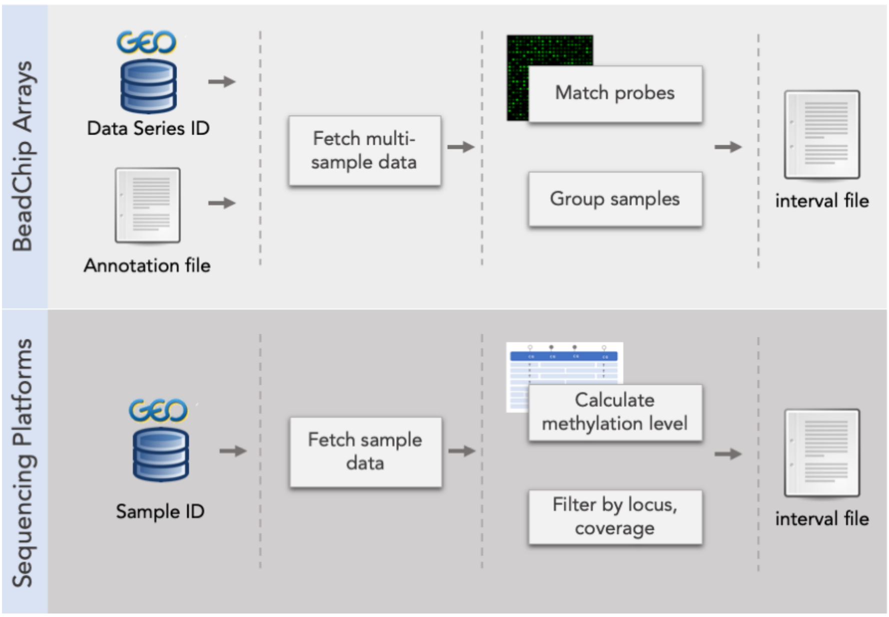


**Figure S5.** Preprocessing steps of DNA methylation data produced by BeadChip array (top) and sequencing platforms (bottom).

**Table S1.** Frequency of DNA conformational changes introduced at sites of differential methylation in various cancer methylation datasets.

| **GEO ID** | **Cancer Data Series** | **MGW** | | **HelT** | | **ProT** | | **Roll** | |
|---|---|---|---|---|---|---|---|---|---|
| | | **DMS$^+$** | **DMS$^-$** | **DMS$^+$** | **DMS$^-$** | **DMS$^+$** | **DMS$^-$** | **DMS$^+$** | **DMS$^-$** |
| GSE42752 | Colorectal adenocarcinoma | 0.020 | 0.038 | 0 | 0 | 0.020 | 0.002 | 0 | 0 |
| GSE54503 | Hepatocellular carcinoma | 0.081 | 0.058 | 0 | 0 | 0.010 | 0.001 | 0 | 0 |
| GSE85464 | Stomach adenocarcinoma | 0.056 | 0.068 | 0 | 0 | 0 | 0.008 | 0 | 0 |
| GSE25093 | Head & Neck SC carcinoma | 0.060 | 0 | 0 | 0 | 0.048 | 0 | 0 | 0 |
| GSE32866 | Lung adenocarcinoma | 0.0457 | 0.087 | 0 | 0 | 0.017 | 0 | 0 | 0 |
| GSE37754 | Breast cancer | 0.0657 | 0.041 | 0 | 0 | 0 | 0.020 | 0 | 0 |
| GSE26989 | Ovarian cancer | 0.054 | 0.080 | 0 | 0 | 0.029 | 0.018 | 0 | 0 |
| GSE109402 | Medulloblastoma | 0.078 | 0.076 | 0 | 0 | 0.016 | 0.018 | 0 | 0 |
| GSE61441 | Renal cell carcinoma | 0.047 | 0.047 | 0 | 0 | 0 | 0 | 0 | 0 |

**MGW**: Minor Groove Width, **HelT**: Helical Twist, **ProT**: Propeller Twist.
